# Individual Variation in Contagious Yawning Susceptibility Is Highly Stable and Largely Unexplained by Empathy or Other Known Factors

**DOI:** 10.1371/journal.pone.0091773

**Published:** 2014-03-14

**Authors:** Alex J. Bartholomew, Elizabeth T. Cirulli

**Affiliations:** Center for Human Genome Variation, Duke University School of Medicine, Durham, North Carolina, United States of America; CNR, Italy

## Abstract

The contagious aspect of yawning is a well-known phenomenon that exhibits variation in the human population. Despite the observed variation, few studies have addressed its intra-individual reliability or the factors modulating differences in the susceptibility of healthy volunteers. Due to its obvious biological basis and impairment in diseases like autism and schizophrenia, a better understanding of this trait could lead to novel insights into these conditions and the general biological functioning of humans. We administered 328 participants a 3-minute yawning video stimulus, a cognitive battery, and a comprehensive questionnaire that included measures of empathy, emotional contagion, circadian energy rhythms, and sleepiness. Individual contagious yawning measurements were found to be highly stable across testing sessions, both in a lab setting and if administered remotely online, confirming that certain healthy individuals are less susceptible to contagious yawns than are others. Additionally, most individuals who failed to contagiously yawn in our study were not simply suppressing their reaction, as they reported not even feeling like yawning in response to the stimulus. In contrast to previous studies indicating that empathy, time of day, or intelligence may influence contagious yawning susceptibility, we found no influence of these variables once accounting for the age of the participant. Participants were less likely to show contagious yawning as their age increased, even when restricting to ages of less than 40 years. However, age was only able to explain 8% of the variability in the contagious yawn response. The vast majority of the variability in this extremely stable trait remained unexplained, suggesting that studies of its inheritance are warranted.

## Introduction

Spontaneous yawning, which occurs more frequently when one is bored or tired, is a deeply rooted, phylogenetic trait that is widespread among vertebrates [1]. In contrast, contagious yawning, which can be triggered in response to hearing, seeing, reading, or thinking about yawning [2]–[4], has only been definitively demonstrated in humans and chimpanzees[3], [5]. The ability to yawn spontaneously begins in humans in utero by 20 weeks of gestation, but contagious yawning does not reliably develop in humans or chimps until childhood [6]–[9].

While much speculative theory has gone into understanding the primary function of yawning, no scholarly consensus has been reached or substantiated. Theories range markedly from a thermoregulatory function, i.e., cooling of the brain and increased oxygen consumption, to behavioral synchronization and communication[10], [11]. The contagious aspect of yawning remains a well-known yet poorly understood phenomenon despite the ability to induce yawning in a laboratory setting from finite stimuli, efforts to identify the underlying neural mechanism, and reported associations with empathy.

Evidence for the role of empathy in contagious yawning spans disciplines and has lent support to the empathetic modeling hypothesis[12]. Studies have found susceptibility to contagious yawning to be correlated with empathic aspects like faux pas theory of mind tasks, self-face recognition, and scores on standardized empathy scales [2], [12], [13]. Intriguingly, patients with either autism spectrum disorder or schizophrenia, both of which exhibit impaired social resonance, demonstrate reduced contagious yawning despite spontaneous yawning remaining intact [13]–[15]. Further support for the role of empathy stems from a longitudinal behavioral study demonstrating a positively modulated contagious yawning frequency and latency along the following cline of increasing social bond: stranger→acquaintance→friend→kin[16]. An experiment in chimpanzees, who display at least basic levels of empathy, furthered this finding by demonstrating increased contagious yawning in response to in-group, compared to out-group, yawners[17]–[19].

Neuroimaging studies have also provided support for the role of empathy in this trait. Despite divergent reports on the recruitment of the human motor neuron system (MNS), there is general consensus that contagious yawning recruits the neural network involved in cognitive empathy[2], [20]–[22]. The MNS may allow for shared emotional and physiological states based on motor patterns [23] and has been previously demonstrated to be more active in empathic individuals[24]. By evaluating unique patterns of activation during contagious yawning, it has also been demonstrated that structures implicated in self-processing and mentalizing, such as cortical midline structures, are recruited during the contagious yawning response[20].

In controlled studies, approximately 40–60% of healthy volunteers yawn in response to a yawn stimulus [3], [4], [12]. Despite this variability, relatively little is known about factors that may influence individual susceptibility to contagious yawning beyond empathy. Purported associations have additionally been made with subjective measures of intelligence, time of day, and climate conditions [7], [25], [26]. However, studies with larger sample sizes have generally not assessed multiple factors simultaneously and have been limited in scope. Additionally, the effect of being observed is inhibitory to contagious yawning[27], [28], which has made studying this trait in a more naturalistic setting a possibly ideal, yet underexplored approach. In particular, no studies have yet assessed whether susceptibility to contagious yawning remains stable when participants are tested both in a laboratory setting and in an uncontrolled setting outside of the laboratory. Only one study has ever assessed whether an individual's susceptibility is stable from one laboratory-based testing session to the next[3].

Here, we aim to better define the role of various factors in susceptibility to contagious yawning by systematically assessing the effect of basic demographics, testing conditions, empathy, cognitive performance, time of day, and other variables on the response of healthy controls to a brief contagious yawning video stimulus. We also aim to define the stability of susceptibility to contagious yawning using our developed yawning stimulus in a laboratory and natural setting. Our overall, long-term goal in characterizing variability in this trait is to create a novel viewpoint into the pathways behind human diseases like schizophrenia and autism, as well as general human functioning, by identifying the genetic basis of normal variation in this genetically understudied, yet clearly biological, trait. The presented work represents the most comprehensive characterization of factors influencing contagious yawning to date.

## Materials and Methods

### Ethics and participants

The Duke University Institutional Review Board approved all procedures and all participants gave written informed consent (IRB# 6828;12268).

Participants (n  =  328) were enrolled as part of the Duke Genetics of Cognition and Other Normal Variation study [29], [30]. All volunteers were included in the study, but the cohort was enriched for young university students due to our location (mean age  =  32.0, range 18–83, standard deviation  =  15.7). Participants completed the tasks in order of presentation below. A more comprehensive description of the participants is reported in Table 1.

**Table 1:** Participant Demographics.

Variable	Mean (SD) or Count %	n
Age in years	32.19 (15.06)	328
Ancestry		328
European	202 (61.6%)	
African	63 (19.2%)	
East Asian	28 (8.5%)	
Other	35 (10.7%)	
Sex		328
Male	108 (32.9%)	
Female	220 (67.1%)	
Education		328
Years of education	15.75 (2.33)	
Current student	177 (54.0%)	
CIRENS	0.31 (1.25)	319
IRI		202
Fantasy	15.52 (5.65)	
Empathic Concern	18.99 (4.39)	
Perspective Taking	17.41 (4.58)	
Personal Distress	10.39 (4.77)	
Emotional Contagion	42.20 (5.64)	128
Location		328
In-lab	199 (60.7%)	
Off-site	129 (39.3%)	
Current sleepiness	2.3 (0.93)	328

*Standard deviation (SD), sample size (n), Circadian Energy Scale (CIRENS), Interpersonal Reactivity Index (IRI).

### Cognitive test

All participants took a brief battery of standardized, well-known cognitive tests assessing diverse areas of cognition [29], [30]_ENREF_31. As previously described, principal component analysis was performed on the participants' scores to determine their overall performance. The first principal component (PC1) explained 49.8% of the total variation in test scores and received approximately equal loadings from all tests. It was therefore taken as a measure of overall cognitive performance on the battery and can be considered a proxy for general intelligence.

### Questionnaire

All participants took an extensive demographic survey that asked them for information including ethnicity and education[30]; participants also took the questionnaires described below.

#### Sleep

The Circadian Energy Scale (CIRENS) is a two-question chronotype measure based on self-report energy levels throughout the day that is strongly correlated (r  =  −.70, *p* < 0.001) with the Horne and Östberg Morningness-Eveningness Scale (MEQ) [31]. Participants (n = 319) described their energy level (very low, low, moderate, high, or very high, scored 1 to 5) in the morning and evening. The difference between the evening score and morning score determined the overall chronotype score, ranging from −4(most marked morning preference) to +4(most marked evening preference). It has previously been shown that differences between chronotypes, or sleep-wake rhythms, affect yawning susceptibility [32].

The Epworth Sleepiness Scale (ESS) is a self-report measure designed to indicate a participant's daytime sleepiness [33]. It asks participants to rate their probability of falling asleep (0 = no chance of dozing to 3 = high chance of dozing) during eight relatively common, daily events. The summation of the eight responses indicate whether a participant is normal (<10), borderline (10–11), or abnormal (12–24). The complete Epworth Sleepiness scale was only collected for the first 266 participants. After this point, answers to only 4 of the 8 responses were collected because the additional questions added little information; r  =  .89 between the score on the original scale and score on the abbreviated scale.

#### Empathy

The Interpersonal Reactivity Index (IRI) is currently one of the most widely used measures of dispositional empathy [34]. It contains two cognitive empathy subscales: perspective taking (PT) and fantasy (FS), and two measures of affective empathy: empathetic concern (EC) and personal distress (PD).

The Perspective-Taking (PT) scale measures the tendency to spontaneously adopt the psychological view of others; the Fantasy (FS) scale assesses tendencies to transpose oneself imaginatively into the feelings and actions of fictitious characters; the Empathic Concern (EC) scale assesses ‘other-oriented’ feelings of sympathy and concern for unfortunate others; the Personal Distress (PD) scale measures ‘self-oriented’ feelings of personal anxiety and unease in tense interpersonal settings [35].

The IRI presents participants with a variety of situations and associated statements regarding feelings and thoughts. Responses are given on a scale of 1 (*does not describe me well*) to 5 (*describes me very well*) and used to calculate an overall score (ranging from 7 to 35) for each empathetic subset scale. Administration of the IRI subscales (n = 202) were halted after a multivariate analysis indicated that any association seen between contagious yawning and IRI subscales was explained by variation in age.

#### Emotional contagion

The Emotional Contagion (EC) Scale is a 15-item self-report, validated measure used to assess individual differences in susceptibility to automatically mimic the emotions of others [36]. Administration of the Emotional Contagion scale was dropped after administration to the first 128 participants due to a clear lack of effect on contagious yawning susceptibility.

#### Contagious yawning

Prior to the start of the video, participants read a brief description of both the characteristics of contagious yawning and the forthcoming video. Participants also rated their overall perceived susceptibility to contagious yawning prior to watching and their current level of sleepiness (0 = energetic to 4 = very tired). The time of day the stimulus was viewed was also recorded.

### Video stimulus

The contagious yawning stimulus is a 183-second video created (Final Cut Pro 7.0.3) using a compilation of yawning faces from the public domain, including video clips (n = 17, mean duration = 7.24 s) and still images (n = 4, shown for 4 s each). Individual stimuli were selected based on perceived naturalness of the yawn. Individuals within the video represent a wide range of ages, from infant to elderly, and cumulatively present yawns from multiple angles. Most individuals within the stimulus are of European ethnicity, though Asian and African-American individuals are also represented; there is an approximately equal distribution of males and females. Stimuli were separated with a 2 s intertrial interval (ITI) where a black screen was shown. A 1 s fade out-fade in was used to transition from ITI to each stimulus.

Stimuli were presented in the lab using the Psychophysics Toolbox extensions[37], [38] in Matlab(2012a), while the video stimulus for off-site participants was temporarily hosted on Youtube. Prior to the start of the video, participants taking the test remotely were instructed to make sure that they were alone, and participants in the lab were left alone in a testing room[27]. The participant was instructed to keep track of the number of times they yawned by clicking an automated counter button[39]. After the video, the participant was then asked how often they felt like yawning (0 = never felt like yawning to 4 = pretty much the entire time) throughout the entire video and whether or not they were alone when viewing from an off-site location.

### Off-site and in-lab testing

Most participants took the questionnaire and watched the yawning video as part of the same lab session where they underwent cognitive testing. However, some participants had taken the cognitive test prior to our beginning the contagious yawning study; these participants were contacted via e-mail to complete the questionnaire and yawning test remotely online, outside of the lab. The questionnaire and yawning test were therefore completed off-site by 129 participants, while the other 199 participants completed these tasks in the lab.

### Repeat sessions

To determine the reliability of the measurements, we contacted the participants to watch the yawning video stimulus a second time. Of the 328 participants, 79 viewed the video twice off-site (mean  =  73.6 days between sessions, SD  =  12.2), and an additional 50 participants re-watched the video off-site after completing the measure once in the laboratory setting (mean  =  85.8 days between sessions, SD  =  20.2).

### Data analysis

All statistical analyses were completed in STATA[40] with the exception of testing whether the correlation between the number of yawns at each session differed between the off-site/off-site and in-lab/off-site repeat sessions, for which we used a Fisher's r-to-z transformation[41]. One participant was greater than four standard deviations away from the mean (n = 15 yawns). Multivariate regression analyses were performed both with and without the outlier included to ensure no impact on the outcomes of the study.

#### Binary analysis

**Figure 1:**
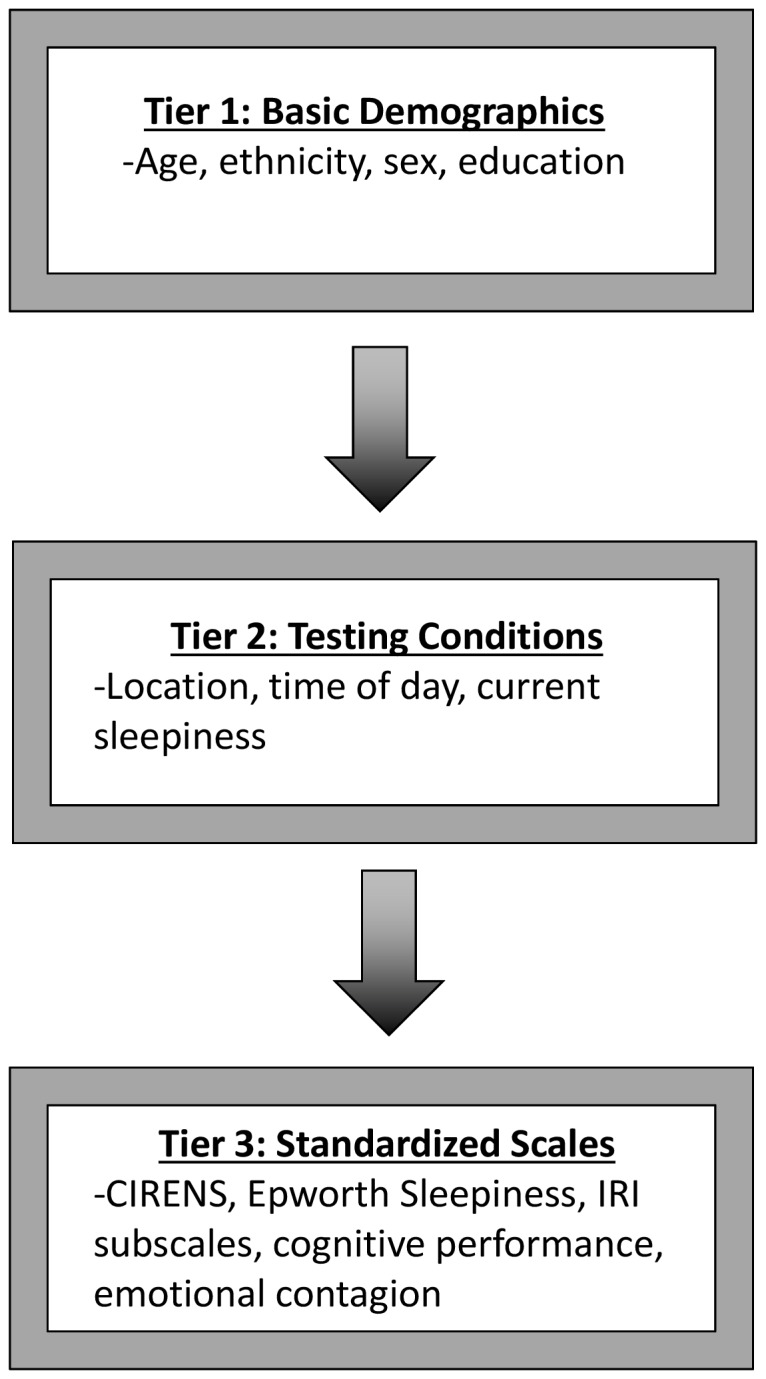
Flow chart of analysis procedure. Regression analyses were performed in three tiers: Tier 1 tested basic demographics as covariates, Tier 2 added testing conditions, and Tier 3 included measurements for standardized scales.

**Figure 2:**
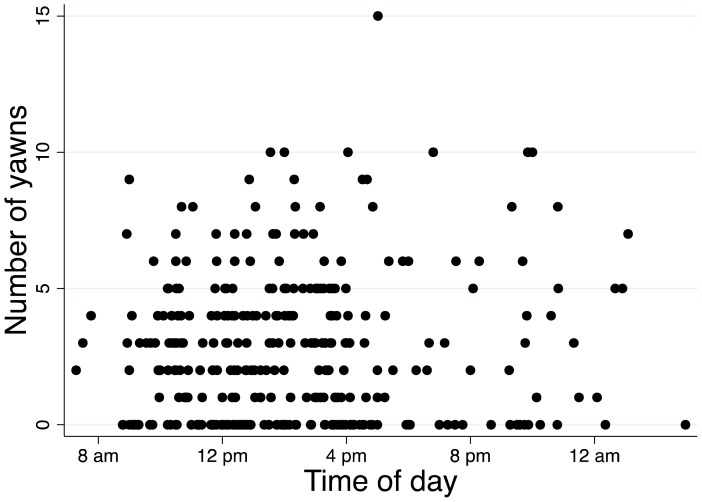
Yawns produced throughout the day. Times after 12:00am were considered to be very late evening. With this definition, the earliest time observed was 7:17am, and the latest was 2:56am.

Participants were grouped into one of two categories: those who contagiously yawned at least one time and those who did not. Stepwise logistic forward regression analyses were performed using a p-value cutoff for inclusion into the model of 0.01. The analysis was performed in three tiers as shown in Figure 1. In Tier 1, the regression model was built using basic demographics as potential covariates. Education was coded as years of education plus a dummy variable for whether they were currently a student, with non-students as the baseline. For Tier 2, those covariates that contributed to the first tier with *p* < .01 were kept in the model, and testing conditions were added as potential covariates. Time of day was analyzed by two methods: as a quantitative representation of the number of minutes in a day (Figure 2), with times between midnight and 3am counting as late night instead of early morning, and as a set of dummy variables corresponding to two-hour time bins throughout the day with additional bins to group times before 10am and after 10pm. For Tier 3, again, those from the previous tier with *p* < .01 were kept, and new potential covariates of performance on standardized scales were added. The pseudo r^2^ values reported here are McFadden's values as output by STATA and were interpreted as generally indicating the amount of variation in susceptibility to contagious yawning explained by the overall model.

#### Quantitative analysis

To examine factors affecting normal variation in contagious yawners (n = 222), we eliminated those who did not yawn in response to the video. The number of yawns was transformed into a normally distributed trait using a Box Cox transformation of ((((yawns∧0.2722135)-1)/0.2722135); Shapiro-Wilk's *p* > .01 after transformation). We then used a linear regression model using the same process as described above for stepwise logistic regression to identify potential covariates influencing this trait.

## Results

**Figure 3:**
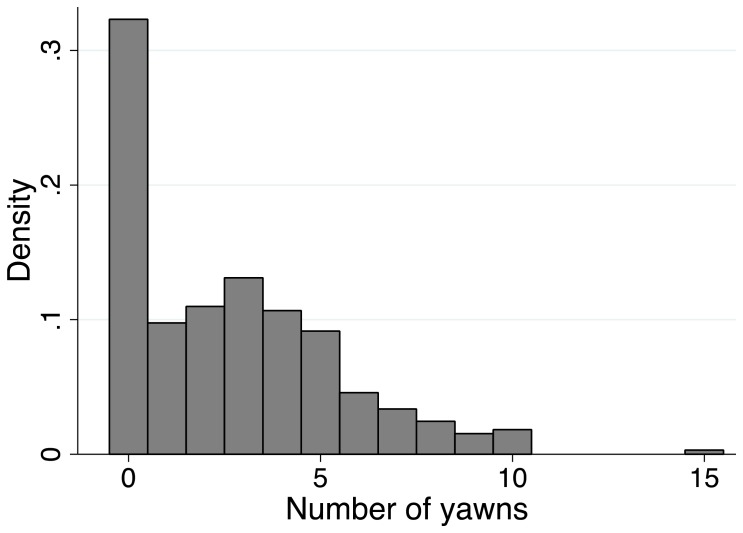
Yawn distribution. This histogram shows the range of zero to fifteen yawns during the 3-minute video stimulus.

Of the 328 participants, 222 contagiously yawned at least once (67.7%), with a range of 0–15 yawns (mean  =  2.66, SD  =  2.68; mean of yawners  =  3.94, SD  =  2.38; Figure 3). Although participants who took the test remotely were instructed to make sure they were alone to avoid yawning inhibition, 14 participants had additional people in the room while they viewed the video. We did not find that the presence of others in the room had any statistically significant effect on whether or not participants exhibited contagious yawning (Fisher's exact *p*  =  .245).

### Binary analysis

For the primary analysis, we focused on whether or not the participants yawned in response to the video stimulus. We investigated the relationship between 15 variables and whether or not the participants contagiously yawned using a tiered approach as follows.

#### Tier 1: Basic demographics

The logistic regression analysis design is shown in Figure 1. Only age (beta  =  −.044, standard error  =  .008, *p* < .001) was a significant predictor of whether or not one was susceptible to contagious yawning. However, age explained only 7.8% of the variation in susceptibility, as indicated by pseudo r^2^ values.

#### Tier 2: Testing conditions

All yawning results were captured concurrently with information about current sleepiness of the participant at the time of testing and the time of day taken; we also considered the location of testing (in-lab or off-site). None of the testing condition variables were found to influence the existing model from Tier 1.

#### Tier 3: Measurements for standardized scales

Empathy (IRI), usual sleepiness level, cognitive performance, emotional contagion, and a chronotype measure (CIRENS) were included as potential covariates along with the participant's age. Due to some traits only being measured in a subset of the sample (Emotional contagion = 128, IRI subscales = 202, CIRENS = 319), the sample size was smaller for this tier. Whether analysis of Tier 3 was restricted to the 128 participants with all measures, the 202 participants who were only missing emotional contagion scores or the 290 who had been measured for just CIRENS, abbreviated Epworth Sleepiness and cognitive performance, none of the Tier 3 variables were found to influence the models from Tier 1 and Tier 2.

### Quantitative analysis

The quantitative analysis investigated differences among the participants who contagiously yawned by excluding the non-yawners. In this multivariate linear regression analysis that used the three-tier approach as described above, no variables had a significant influence on the trait.

### Self-assessment

Participants rated their overall perceived susceptibility to contagious yawning prior to watching the stimulus video. Perceived susceptibility was correlated with whether participants yawned (*p*  =  0.002; beta  =  0.478; pseudo r^2^  =  0.024), and was positively associated with the number of yawns exhibited by yawners (*p* < 0.001; beta  =  0.280; r^2^  =  0.065). Scores for perceived yawning susceptibility were enriched for a perception of being susceptible, with 94% of participants indicating that they sometimes, often, or usually yawned when they saw someone else yawn; in contrast, only 68% of participants actually yawned during this study.

After the video, participants rated how often they felt like yawning during the video. This measure was strongly positively correlated with both whether they yawned (*p* < 0.001; beta  =  2.47; pseudo r^2^  =  0.331) and the range of yawns exhibited by yawners (*p* < 0.001; beta  =  0.644; r^2^  =  0.312). Fifty-nine percent of the participants who did not yawn reported that they did not feel like yawning during the video, as opposed to one participant who indicated they never felt like yawning, despite yawning once during the video.

### Reliability of contagious yawning susceptibility

**Figure 4:**
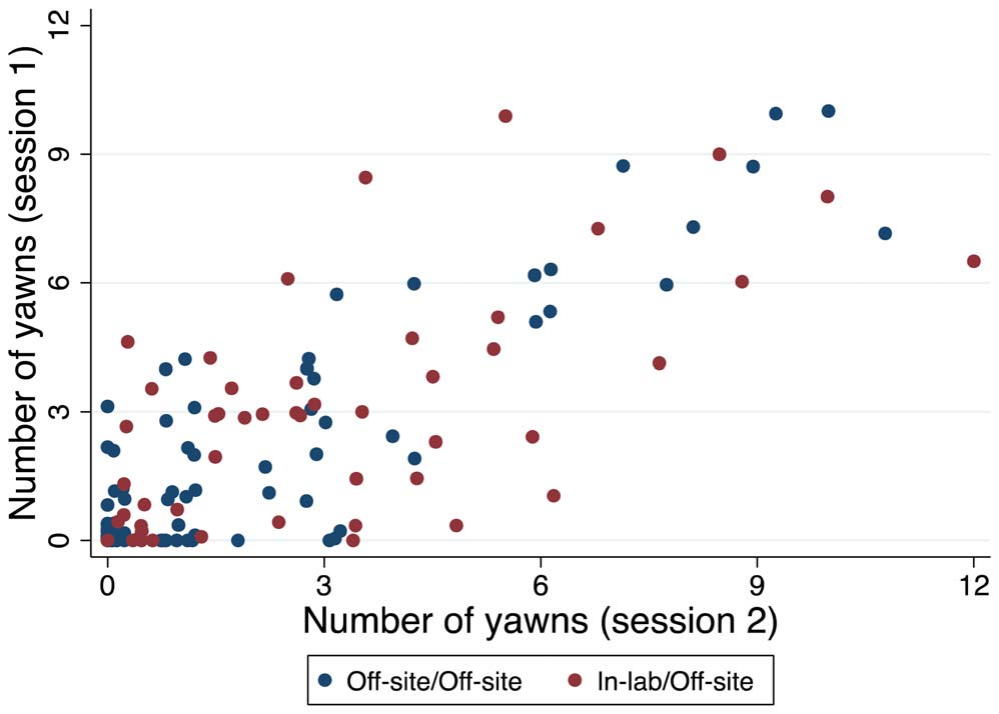
Correlation between the yawns observed at the first and second testing session. Data were jittered in this plot to give a feel for how many observations make up each point (Pearson's r  =  0.80, *p* < 0.001).

To measure the stability of susceptibility to contagious yawning, 79 participants watched the yawn video twice off-site, and 50 participants watched the yawning video once in the lab and then re-took the measure remotely. Of these 129 participants, 78.3% remained in the same binary yawn category between sessions, and a two-sided t-test confirmed no significant difference in the raw change in the number of yawns based on the testing locations. We obtained a Pearson's r of 0.80 between the two testing sessions (Figure 4), although the correlation for the off-site repeat sessions (0.87) was significantly higher (*p*  =  0.007) than was that for the in-lab followed by off-site repeat session (0.68).

## Discussion

We assessed the impact of multiple factors on contagious yawning susceptibility in a group of 328 healthy volunteers who exhibited contagious yawning frequencies that were similar to those from the previous literature [3], [4]. Our results reveal that variables like empathy, tiredness, and Circadian preference have little effect on contagious yawning susceptibility and that the contagious yawning response of individuals is stable over a two-month period, whether they are tested in the lab, or off-site via an online test.

**Figure 5:**
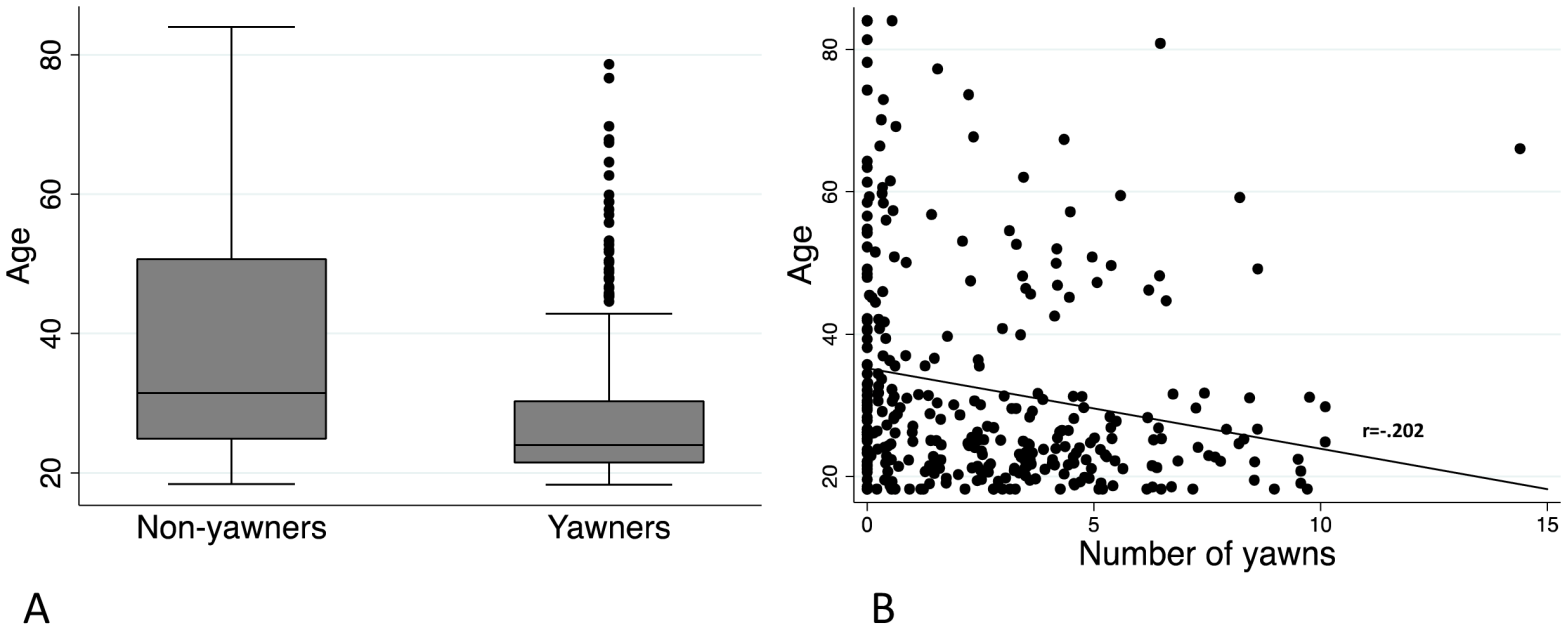
Age and contagious yawning. a) A box-and-whisker plot showing the age distribution for those who did and did not yawn during the test and b) the correlation between age and the raw number of yawns elicited (Pearson's r  =  −.202, *p* < 0.001).

The results demonstrate that the age of the participant was the only variable with a significant influence on whether or not they yawned. This association was not simply the result of the wide range of ages assessed here (Figure 5); even when restricting to participants aged below 40, age was still the only significant predictor of susceptibility to contagious yawning. Despite this strong association, age was only able to explain 8% of the variation in the yawning response, leaving the majority of variation unexplained by any known factors. Interestingly, a reduction in yawning frequency has been previously demonstrated in aged individuals, though never previously in a contagious context [42].

Our results are in contrast to previous studies, which have identified correlations between yawning susceptibility and empathic abilities, time of day, and subjective measures of intelligence [7], [12], [13], [25]. The IRI Fantasy, which gauges one's capacity for cognitive empathy and was previously demonstrated to influence susceptibility to contagious yawning in a sample of 45 healthy controls [13], was not a significant predictor of susceptibility in our study when taking age into account, despite the general viewpoint that contagious yawning must be a product of empathy [12], [14], [43], [44]. Our participants were measured for several aspects of empathy, including all four portions of the well-known IRI and an established emotional contagion test. While the sample size for the empathy scales was smaller than the sample size for the rest of our study, the number of participants measured was still larger than the majority of previous studies on contagious yawning and would have been more than sufficient to pick up a strong effect. This lack of association suggests that contagious yawning is not simply a product of one's capacity for empathy.

When examining variables individually in univariate logistic regression analyses, we did identify associations between contagious yawning susceptibility and education, whether one was currently a student, cognitive performance, Circadian preference (CIRENS), empathy (IRI Fantasy subscale), and current tiredness. However, these variables were all even more strongly associated with the age of the participant and were no longer significantly associated with contagious yawning susceptibility when taking age into account. While the associations between these factors and age were largely already known to exist [45]–[48], the reason for the association between contagious yawning and age remains unknown, offering a direction for future exploration. Possible explanations for this strong, inverse association could include decreased attention to the stimulus with age, a reduced connection to the yawners in the video due to use of technology, or a general decline in susceptibility to contagious yawning as we age.

To our knowledge, only one previous study of 37 participants has measured the test-retest reliability of a contagious yawning susceptibility test [3]. Our work demonstrates high reliability in individual yawn responses to a 3-minute yawn stimulus video, whether it is taken twice outside the lab or once in the lab and then a second time outside the lab. While the correlation between the two test sessions was lower in the in-lab/off-site repeat session, this is not unexpected given the change in testing conditions. Furthermore, both sets of correlations for these repeat sessions are comparable to those of the previous work, despite the difference in our testing locations and even though our studies differed markedly in length, stimulus type and sample size. Our results provide new evidence for the stability of contagious yawning susceptibility across testing sessions and locations and indicate that constant differences exist between healthy controls in their susceptibility.

This study does have some limitations. It is worth noting that our goal was not to describe the frequency of yawns in response to a specific video, but rather to reliably measure differences between individuals in their response to a short, standardized yawn stimulus. We therefore make no claims about the precise frequency of contagious yawns elicited by the video stimulus. In addition, participants were primed with a brief description of contagious yawning, which may have contributed to the slightly elevated percentage of contagious yawners in our population; it is also possible that some recorded yawns were actually spontaneous yawns. We did not directly observe the participants, in contrast to many previous contagious yawning studies. This method was chosen because the high word of mouth advertisement about our study makes secretive procedures like surreptitious observation difficult to maintain for all participants. The strong test-retest correlation demonstrates that our method is valid and is in accordance with a previous study showing that participants were able to accurately record their own yawns while being secretly videotaped [39]. Additionally, we employed several self-report scales that may not accurately reflect, for example, the true empathy or circadian preference of the participant. Nonetheless, these scales are either current standards in the field or are well correlated with them, providing us with the best representation of these traits that is available with a brief questionnaire. Finally, we interpreted pseudo r^2^ values from the logistic regression models as approximations of the amount of variation in contagious yawning explained by the variables investigated, although these values cannot be interpreted as reliably as can the traditional r^2^ values from linear regression models.

Despite these limitations, our work clearly demonstrates the stability of intra-individual variation in susceptibility to contagious yawning, a significant negative correlation between age and the contagious yawning response, and the inability of any known variables to explain the vast majority of variation in contagious yawn responses. This extensive, unexplained, and highly replicable variation between individuals in their susceptibility suggests the existence of an underlying genetic influence and warrants future studies assessing the inheritance of this unique trait.
